# Dynamic preload indicators decrease when the abdomen is opened

**DOI:** 10.1186/1471-2253-14-90

**Published:** 2014-10-14

**Authors:** Martijn van Lavieren, Jeroen Veelenturf, Charlotte Hofhuizen, Marion van der Kolk, Johannes van der Hoeven, Peter Pickkers, Joris Lemson, Benno Lansdorp

**Affiliations:** University of Twente, MIRA - Institute for Biomedical Technology and Technical Medicine, PO box 217, Enschede, 7500 AE The Netherlands; Department of Anaesthesiology, Radboud university medical center, PO Box 9101, Nijmegen, 6500 HB The Netherlands; Department of Surgery, Radboud university medical center, PO Box 9101, Nijmegen, 6500 HB The Netherlands; Department of Intensive Care, Radboud university medical center, PO Box 9101, Nijmegen, 6500 HB The Netherlands

## Abstract

**Background:**

Optimizing cardiac stroke volume during major surgery is of interest to many as a therapeutic target to decrease the incidence of postoperative complications. Because dynamic preload indicators are strongly correlated with stroke volume, it is suggested that these indices can be used for goal directed fluid therapy. However, threshold values of these indicators depend on many factors that are influenced by surgery, including opening of the abdomen. The aim of this study was therefore to assess the effect of opening the abdomen on arterial pressure variations in patients undergoing abdominal surgery.

**Methods:**

Blood pressure and bladder pressure were continuously recorded just before and after opening of the abdomen in patients undergoing elective laparotomy. Based on waveform analysis of the non-invasively derived blood pressure, the stroke volume index, pulse pressure variation (PPV) and stroke volume variation (SVV) were calculated off-line.

**Results:**

Thirteen patients were included. After opening the abdomen, PPV and SVV decreased from 11.5 ± 5.8% to 6.4 ± 2.9% (p < 0.005, a relative decrease of 40 ± 19%) and 12.7 ± 6.1% to 4.8 ± 1.6% (p < 0.05, a relative decrease of 53 ± 26%), respectively. Although mean arterial pressure and stroke volume index tended to increase (41 ± 6 versus 45 ± 4 ml/min/m2, p = 0.14 and 41 ± 6 versus 45 ± 4 ml/min/m2, p = 0.05), and heart rate tended to decrease (73 ± 15 versus 68 ± 11 1/min, p = 0.05), no significant change was found. No significant change was found in respiratory parameter (tidal volume, respiratory rate or inspiratory pressure; p = 0.36, 0.34 and 0.17, respectively) or bladder pressure (6.0 ± 3.7 versus 5.6 ± 2.7 mmHg, p = 0.6) either.

**Conclusions:**

Opening of the abdomen decreases PPV and SVV. During goal directed therapy, current thresholds for fluid responsiveness should be changed accordingly.

## Background

Optimizing cardiac stroke volume during major surgery is of interest to many as a therapeutic target to decrease postoperative complications and the length of stay in the ICU [1–3].

Dynamic preload indicators, like stroke volume variation (SVV) and pulse pressure variation (PPV), are reliable predictors of fluid responsiveness during mechanical ventilation [4]. Therefore, it has been suggested that these indicators should be optimized during major surgery by goal directed fluid therapy [5].

Dynamic preload indicators are the result of the concerted effect of the swings in intrathoracic pressure during mechanical ventilation [6]. The amplitude of the intrathoracic pressure depends, in case of positive pressure ventilation, upon the space occupied by the inflated lungs within the thoracic compartment, hence tidal volume, and the compliance of the thoracic cavity [7]. Because the diaphragm is part of the thoracic cavity, the resistance of the diaphragm to change shape, contributes to the chest wall compliance. In turn, this resistance of the diaphragm is influenced by the abdominal pressure (Pab), which acts as an opposing force to the diaphragmatic descent during mechanical inspiration. An increase in Pab therefore results in decreased chest wall compliance and more pronounced arterial pressure variations [8]. For this reason, the use of higher threshold values has been suggested for the prediction of fluid responsiveness in patients with increased Pab [9]. However, the influence of a decreased abdominal pressure on dynamic preload indicators is unknown.

We hypothesized that by opening of the abdominal compartment, the chest wall compliance will increase and dynamic preload indicators will be decreased in magnitude. This would imply that currently used threshold values for the prediction of fluid responsiveness are not applicable during open abdomen surgical procedures. The aim of this study was therefore to assess the effects of opening of the abdominal cavity on dynamic preload indicators.

## Methods

### Patients

Thirteen patients on controlled mechanical ventilation were studied during elective abdominal surgery requiring laparotomy. Because of the observational and non-invasive character of this study, the local medical ethics board (Ethics committee region Arnhem Nijmegen) waived the need for informed consent. Exclusion criteria consisted of a Body Mass Index (BMI) >35 kg/m [2], recent (<2 months) bladder surgery or trauma and any cardiac arrhythmias. Patients were also excluded when administration of epidural anaesthetics or fluid resuscitation was needed to maintain hemodynamic stability during the execution of the study protocol.

### Physiological monitoring

Pressure monitoring included non-invasive arterial blood pressure (ABP) and bladder pressure as a measure of abdominal pressure (Pab). The ABP was measured continuously using an inflatable finger cuff in combination with the Nexfin™ Monitor (BMEYE, Amsterdam, The Netherlands) [10]. After insertion of the urinary catheter the empty bladder was filled with 25 ml of 0.9% NaCl solution (Baxter BV, Utrecht, The Netherlands) and subsequently connected to a pressure monitoring set (Edwards Lifesciences LLC, Irvine, California, USA) [11]. Both blood pressure and bladder pressure were recorded on a laptop computer with a sample rate of 200 Hz using an A/D converter (NI USB-6211, National Instruments, Austin, Texas, USA). Cardiac index (CI) and stroke volume index (SVI) were calculated from the ABP using the pulse contour method incorporated in the Nexfin™ Monitor (BMEYE, Amsterdam, The Netherlands) [12, 13]. Pulse pressure variation (PPV) and stroke volume variation (SVV) were calculated offline using Matlab (Matlab R2012a, Mathworks Inc., Natick, MA, USA), according to equation 1 and 2, respectively:
12

in which PPmax/SVImax and PPmin/SVImin were the maximum and minimum pulse pressure/stroke volume index over one breath, and subsequently averaged over 5 consecutive respiratory cycles [14]. All data used in the analysis was visually inspected for artefacts.

### Study protocol

Recording of physiological data started after anaesthetic induction. Operating procedures were performed according to standard clinical practice. After opening the abdominal compartment, no retractors were placed during the measurement to avoid strain induced by the retractors on the abdominal tissue. The urinary catheter was clamped shortly after insertion after all urine had drained from the bladder. After clamping the catheter, a 21G needle connected to a pressure monitoring set was inserted and secured in the entry point of the catheter. Recording of the abdominal pressure started one minute after the priming of the bladder by injecting 25 mL of 0.9% NaCl. ABP, Pab and respiratory parameters were recorded for one minute just before opening of the abdominal compartment and one minute immediately after. Patients were ventilated with (pressure regulated) volume-controlled ventilation in order to maintain the same tidal volume before and after opening of the abdomen (including the fascia) since tidal volume is the main determinant of changes in intrathoracic pressure.

### Anaesthetics

Anaesthesia was induced with propofol 1–2.5 mg kg^-1^ and sufentanil 10–50 μg. Endotracheal intubation was facilitated with neuromuscular blockade established with rocuronium 0.6-1.0 mg kg^-1^. Anaesthesia was maintained with isoflurane 0.6-1.1% or sevoflurane 1.1-1.6% applied with a mixture of air and oxygen. When an epidural catheter was placed, the position was verified with a test dose of 3 ml lidocaine 2% with 5 μg ml^-1^ epinephrine. Further epidural local anaesthetics were not administered until after the measurements.

### Statistical analysis

Sharipo-Wilk tests were performed to verify a Gaussian distribution of the data. All results are displayed as mean and standard deviation (SD), unless stated otherwise. A paired student t-test is performed to determine the statistical significance of the change in parameters before and after opening of the abdomen. Statistical analysis was performed using GraphPad Prism version 5.00 for Windows (GraphPad Software, San Diego, CA, USA), and a two-sided p-value of <0.05 was considered significant.

## Results

The study population consisted of thirteen patients. In three patients SVV could not be determined due to technical difficulties with the algorithm that derives beat-to-beat stroke volume from the arterial blood pressure signal. All data were normally distributed. Patient characteristics are presented in Table 1.

**Table 1:** **Patient characteristics**

Patients (male/female)	6/7
Age (yr)	54 ± 13
Weight (kg)	81 ± 16
BMI (kg m^-2^)	27 ± 4
Ventilatory mode	(Pressure Regulated) Volume Controlled mode

Data expressed as mean ± SD.

**Figure 1:**
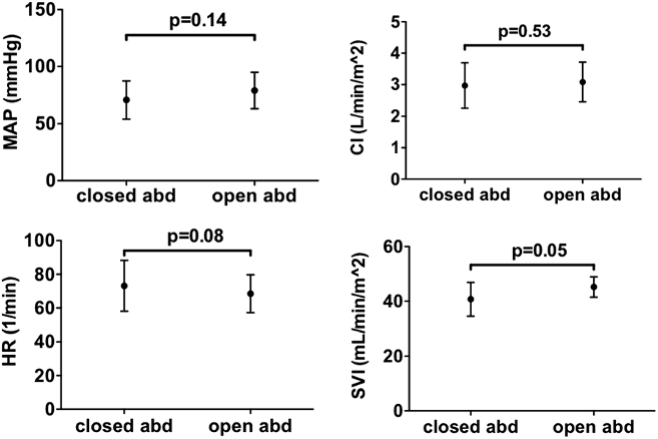
**Comparison of main hemodynamic variables during a closed abdomen and opened abdomen.** MAP = mean arterial pressure, CI = Cardiac Index, HR = heart rate, SVI = Stroke Volume Index, p-values were not significant and mentioned in the figure.

Table 2 shows the ventilatory parameters (tidal volume [ml/kg predicted body weight]), respiratory rate, peak pressure and positive end expiratory pressure) before and after opening of the abdomen. No significant differences were found as a result of the opening of the abdomen. Although heart rate, mean arterial pressure, SVI or CI tended to change, no significant differences were found (see Figure 1). There were also no relevant changes in infusion rate of anaesthetics (sevoflurane, isoflurane, sufentanil, ephidrine or phenylephrine).

**Figure 2:**
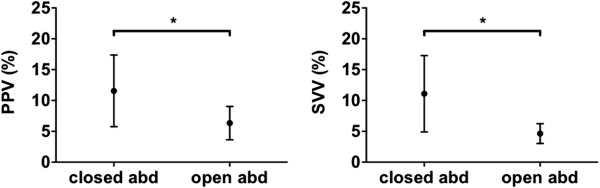
**Comparison of waveform derived preload indicators at closed abdomen and at opened abdomen.** PPV = pulse pressure variation, SVV = stroke volume variation, * = p < 0.01.

As a result of the opening of the abdomen, the PPV significantly (p < 0.005) decreased from 11.5 ± 5.8% to 6.4 ± 2.9%, a relative decrease of 40 ± 19%, see Figure 2. The SVV also decreased significantly (p < 0.05) from 12.7 ± 6.1% to 4.8 ± 1.6% (a relative decrease of 53 ± 26%).

**Table 2:** **Ventilatory parameters just before and after opening of the abdomen**

	Closed abdomen	Open abdomen	P-value
Respiratory rate (1 min^-1^)	12.1 ± 1.0	12.1 ± 1.0	0.34
Tidal volume (ml kg^-1^)	7.6 ± 1.2	7.3 ± 0.7	0.36
PEEP (cmH_2_O)	4.5 ± 1.7	4.7 ± 1.5	0.32
Peak inspiratory pressure (cmH_2_O)	16.6 ± 2.6	15.9 ± 2.6	0.17

PEEP: positive end expiratory pressure. Data expressed as mean ± SD.

Opening of the abdomen did not significantly change mean bladder pressure (mean pressure of 6.0 ± 3.7 versus 5.6 ± 2.7 mmHg, p = 0.6).

## Discussion

This study shows that the magnitude of both PPV and SVV decrease upon opening the abdomen, emphasizing that caution is advised when using these dynamic preload indicators for goal directed fluid therapy during open abdominal surgery.

Dynamic preload indicators accurately predict fluid responsiveness under stable and controlled physiological conditions (e.g. tidal volume >8 ml/kg, no arrhythmias and HR to ventilator ratio >3.6), however when applied under ambiguous physiological conditions their predictive value deteriorates [15]. Hence, when applying these indicators in a surgical setting cautiousness is mandated by the fact that patients’ physiology may be affected by administered drugs, anesthesia and surgery. This vigilance required during the application of dynamic preload indicators in the setting of surgery is increasingly recognized, and together with our findings, emphasizes the drawbacks of dynamic preload indicators in clinical practice [16]. When abdominal pressure increases, e.g. due to pneumoperitoneum, dynamic preload indicators increase, subsequently decreasing their predictive accuracy if the currently proposed threshold values would be applied [8, 9]. While several studies have suggested that dynamic preload indicators are still able to predict fluid responsiveness during abdominal surgery to a certain extent [17–20], our findings are concordant with the results of other studies that their predictive value regarding fluid responsiveness might be reduced compared to values obtained on the ICU [4, 21, 22].

Since the tidal volume, the main determinant for the change in intrathoracic pressure [23], did not change, the observed decrease of the dynamic preload indicators could well be attributed to a decrease in pleural pressure amplitude due to the increase in thoracic compliance (e.g. the diaphragm experiences less resistance from the abdominal compartment during inspiration). This increase in thoracic compliance however, did not result in a significant decrease in inspiratory pressure. This is explained by the fact that the main determinant of the airway pressure, lung compliance, did not change in our patients that did not suffer from any serious illness and had no apparent chest wall edema. Sympathetic reflexes triggered by surgical incision could have attributed to our findings, however since no changes in heart rate and blood pressure occurred we considered its effect to be of lesser importance. Our finding that also the bladder pressure was not decreased is likely related to patients being under general anaesthesia, and having received muscle relaxation, when the abdominal pressure is mainly determined by the gravity component of the above-lying structures, and not the compliance of the abdominal compartment. This is also confirmed by the fact that the swings in bladder pressure due to mechanical ventilation were only 0.6 ± 0.2 mmHg during closed abdomen, and that this change was similar in open abdominal situation (0.6 ± 0.3 mmHg, p = 0.74). Another reason might be that we used an indirect method to measure abdominal pressure instead of a direct method. Although this method is considered the gold standard [11], there are some concerns regarding the reliability and reproducibility of this indirect method [24]. Also urine output in between the two measurements could be a reason for the bladder pressure not to decrease. However, since the time in between the two measurements was only around 15 minutes, the influence of this effect was probably negligible.

This study has several limitations that need to be addressed. First, the arterial pressure was not measured by an invasive arterial catheter, but non-invasively using a finger cuff. As this method has an excellent correlation with invasively recorded arterial pressure and the derived dynamic preload predictors [13] as well as tracking the changes in cardiac index [25], it is unlikely that this impairs the validity of our results. Second, several patients received an epidural catheter prior to the surgery and location was verified with a test dose of 3 ml lidocaine 2% with 5 μg ml^-1^ epinephrine. Due to the low dose of lidocaine administered (which will also block a few segments) and the time interval until the measurements (at least 25 minutes), the test dose was assumed not to influence the measurements [26, 27]. We also assumed that vasoactive drugs, administered during surgery, did not influence the dynamic preload indicators, however studies on this subject are scarce [28, 29]. To obviate the prerequisite of hemodynamic equivalence before and after opening of the abdominal compartment, both blood pressure and cardiac index between measurements were compared and found to be similar, legitimating the assumption of an unchanged hemodynamic status in between measurements. Furthermore, the mean tidal volume that was used to ventilate the patients was somewhat less than the tidal volume used in most studies about the predictive value of dynamic indices (8 ml/kg vs. 7.6 and 7.3 ml/kg). This is because nowadays, lower values of tidal volume are preferred [30, 31]. However, based on a recent publication [32] and the fact that this difference was rather small (0.4 and 0.7 ml/kg) the influence of the reduced tidal volume is suggested to be relatively low compared to the influence of the opening of the abdomen. Also, our study is not designed or used to validate or redefine the commonly used thresholds, so the potentially lowered absolute value of the PPV (influenced by the amount of TV) is not likely to influencing our results or conclusions. Finally, the aim of this observational study was to show the influence of opening the abdomen on the value of the dynamic indices. The study was not designed to quantify the impact on the predictive value neither to propose new thresholds because we did not measure the actual fluid responsiveness of our patients. Because of the major impact of opening the abdomen on the value of the dynamic indices, further research is needed in order to quantify and validate thresholds that are corrected for the abdominal condition of the patient.

## Conclusion

In this study we have assessed the effects of opening of the abdominal cavity on dynamic preload indicators. A significant decrease in arterial waveform derived dynamic variables, SVV and PPV, was found after opening the abdominal compartment, indicating an increased risk of false negative predictions for fluid responsiveness if unchanged thresholds would be applied.

## References

[1] Corcoran T, Rhodes JE, Clarke S, Myles PS, Ho KM (2012). Perioperative fluid management strategies in major surgery: a stratified meta-analysis. Anesth Analg.

[2] Dalfino L, Giglio MT, Puntillo F, Marucci M, Brienza N (2011). Haemodynamic goal-directed therapy and postoperative infections: earlier is better. A systematic review and meta-analysis. Crit Care.

[3] Kirov MY, Kuzkov VV, Molnar Z (2010). Perioperative haemodynamic therapy. Curr Opin Crit Care.

[4] Marik PE, Monnet X, Teboul JL (2011). Hemodynamic parameters to guide fluid therapy. Ann Intensive Care.

[5] Hamilton MA, Cecconi M, Rhodes A (2011). A systematic review and meta-analysis on the use of preemptive hemodynamic intervention to improve postoperative outcomes in moderate and high-risk surgical patients. Anesth Analg.

[6] Michard F (2005). Changes in arterial pressure during mechanical ventilation. Anesthesiology.

[7] Novak R, Matuschak GM, Pinsky MR (1988). Effect of positive-pressure ventilatory frequency on regional pleural pressure. J Appl Physiol.

[8] Jacques D, Bendjelid K, Duperret S, Colling J, Piriou V, Viale JP (2011). Pulse pressure variation and stroke volume variation during increased intra-abdominal pressure: an experimental study. Crit Care.

[9] Tavernier B, Robin E (2011). Assessment of fluid responsiveness during increased intra-abdominal pressure: keep the indices, but change the thresholds. Crit Care.

[10] Eeftinck Schattenkerk DW, Van Lieshout JJ, van den Meiracker AH, Wesseling KR, Blanc S, Wieling W, van Montfrans GA, Settels JJ, Wesseling KH, Westerhof BE (2009). Nexfin noninvasive continuous blood pressure validated against Riva-Rocci/Korotkoff. Am J Hypertens.

[11] Cheatham ML, Malbrain ML, Kirkpatrick A, Sugrue M, Parr M, De Waele J, Balogh Z, Leppäniemi A, Olvera C, Ivatury R, D'Amours S, Wendon J, Hillman K (2007). Results from the international conference of experts on intra-abdominal hypertension and abdominal compartment syndrome. II. Recommendations. Intensive Care Med.

[12] Wesseling KH, Smith NT, Nichols WW, Weber H, De Wit B, Beneken JE, Feldman SA, Leigh JM, Spierdijk J (1974). Beat-to-beat cardiac output from the arterial pressure pulse contour. Measurements in Aneasthesia.

[13] Lansdorp B, Ouweneel D, De Keijzer A, Van der Hoeven JG, Lemson J, Pickkers P (2011). Non-invasive measurement of pulse pressure variation and systolic pressure variation using a finger cuff corresponds with intra-arterial measurement. Br J Anaesth.

[14] Kim HK, Pinsky MR (2008). Effect of tidal volume, sampling duration, and cardiac contractility on pulse pressure and stroke volume variation during positive-pressure ventilation. Crit Care Med.

[15] Lansdorp B, Lemson J, Van Putten MJ, De Keijzer A, van der Hoeven JG, Pickkers P (2012). Dynamic indices do not predict volume responsiveness in routine clinical practice. Br J Anaesth.

[16] Yang SY, Shim JK, Song Y, Seo SJ, Kwak YL (2013). Validation of pulse pressure variation and corrected flow time as predictors of fluid responsiveness in patients in the prone position. Br J Anaesth.

[17] Derichard A, Robin E, Tavernier B, Costecalde M, Fleyfel M, Onimus J, Lebuffe G, Chambon JP, Vallet B (2009). Automated pulse pressure and stroke volume variations from radial artery: evaluation during major abdominal surgery. Br J Anaesth.

[18] Lee JY, Park HY, Jung WS, Jo YY, Kwak HJ (2012). Comparative study of pressure- and volume-controlled ventilation on stroke volume variation as a predictor of fluid responsiveness in patients undergoing major abdominal surgery. J Crit Care.

[19] Benes J, Chytra I, Altmann P, Hluchy M, Kasal E, Svitak R, Pradl R, Stepan M (2010). Intraoperative fluid optimization using stroke volume variation in high risk surgical patients: results of prospective randomized study. Crit Care.

[20] Forget P, Lois F, De Kock M (2010). Goal-directed fluid management based on the pulse oximeter-derived pleth variability index reduces lactate levels and improves fluid management. Anesth Analg.

[21] Hoiseth LO, Hoff IE, Skare O, Kirkeboen KA, Landsverk SA (2011). Photoplethysmographic and pulse pressure variations during abdominal surgery. Acta Anaesthesiol Scand.

[22] Gouvea G, Diaz R, Auler L, Toledo R, Martinho JM (2009). Evaluation of the pulse pressure variation index as a predictor of fluid responsiveness during orthotopic liver transplantation. Br J Anaesth.

[23] Jardin F, Genevray B, Brun-Ney D, Bourdarias JP (1985). Influence of lung and chest wall compliances on transmission of airway pressure to the pleural space in critically ill patients. Chest.

[24] Malbrain ML (2004). Different techniques to measure intra-abdominal pressure (IAP): time for a critical re-appraisal. Intensive Care Med.

[25] Truijen J, Van Lieshout JJ, Wesselink WA, Westerhof BE (2012). Noninvasive continuous hemodynamic monitoring. J Clin Monit Comput.

[26] Visser WA, Liem TH, Van Egmond J, Gielen MJ (1998). Extension of sensory blockade after thoracic epidural administration of a test dose of lidocaine at three different levels. Anesth Analg.

[27] Holman SJ, Bosco RR, Kao TC, Mazzilli MA, Dietrich KJ, Rolain RA, Stevens RA (2001). What constitutes an effective but safe initial dose of lidocaine to test a thoracic epidural catheter?. Anesth Analg.

[28] Hadian M, Severyn DA, Pinsky MR (2011). The effects of vasoactive drugs on pulse pressure and stroke volume variation in postoperative ventilated patients. J Crit Care.

[29] Cannesson M, Jian Z, Chen G, Vu TQ, Hatib F (2012). Effects of phenylephrine on cardiac output and venous return depend on the position of the heart on the Frank-Starling relationship. J Appl Physiol (1985).

[30] Girard TD, Bernard GR (2007). Mechanical ventilation in ARDS: a state-of-the-art review. Chest.

[31] Eichacker PQ, Gerstenberger EP, Banks SM, Cui X, Natanson C (2002). Meta-analysis of acute lung injury and acute respiratory distress syndrome trials testing low tidal volumes. Am J Respir Crit Care Med.

[32] Lansdorp B, Hofhuizen C, Van Lavieren M, van Swieten H, Lemson J, van Putten MJ, van der Hoeven JG, Pickkers P (2014). Mechanical ventilation-induced intrathoracic pressure distribution and heart-lung interactions*. Crit Care Med.

[CR33] The pre-publication history for this paper can be accessed here:http://www.biomedcentral.com/1471-2253/14/90/prepub

